# Non-pharmacological prevention of postoperative delirium by occupational therapy teams: A randomized clinical trial

**DOI:** 10.3389/fmed.2023.1099594

**Published:** 2023-02-02

**Authors:** Evelyn A. Alvarez, Veronica A. Rojas, Lorena I. Caipo, Melany M. Galaz, Daniela P. Ponce, Rodrigo G. Gutierrez, Felipe Salech, Eduardo Tobar, Fernando I. Reyes, Rodrigo C. Vergara, Jose I. Egaña, Constanza A. Briceño, Antonello Penna

**Affiliations:** ^1^Terapia Ocupacional, Universidad Central de Chile, Santiago, Chile; ^2^Departamento de Terapia Ocupacional y Ciencia de la Ocupación, Universidad de Chile, Santiago, Chile; ^3^Critical Care Unit, Department of Medicine, Hospital Clínico de la Universidad de Chile, Santiago, Chile; ^4^Centro de Investigación Clínica Avanzada (CICA), Hospital Clínico y Facultad de Medicina de la Universidad de Chile, Santiago, Chile; ^5^Departamento de Anestesiología y Medicina Perioperatoria, Hospital Clínico de la Universidad de Chile, Santiago, Chile; ^6^Sección de Geriatría, Departamento de Medicina, Hospital Clínico de la Universidad de Chile, Santiago, Chile; ^7^Servicio de Anestesiología, Hospital Santiago Oriente Doctor Luis Tisné Brousse, Santiago, Chile; ^8^Núcleo de Bienestar y Desarrollo Humano (NUBIDEH), Centro de Investigación en Educación (CIE-UMCE), Universidad Metropolitana de Ciencias de la Educación, Santiago, Chile; ^9^Facultad de Artes y Educación Física, Departamento de Kinesiología, Universidad Metropolitana de Ciencias de la Educación, Santiago, Chile; ^10^Centro Nacional de Inteligencia Artificial (CENIA), Santiago, Chile

**Keywords:** occupational therapy, postoperative delirium, very elderly, non-pharmacological prevention, cognitive impairment, major non-cardiac surgery

## Abstract

**Background:**

Patients who develop postoperative delirium (POD) have several clinical complications, such as increased morbidity, increased hospital stays, higher hospital costs, cognitive and functional impairment, and higher mortality. POD is a clinical condition preventable by standard non-pharmacological measures An intensive Occupational Therapy (OT) intervention has been shown to be highly effective in preventing delirium in critically ill medical patients, but it is unknown the effect in surgical patients. Thus, we designed a prospective clinical study with the aim to determine whether patients undergoing intervention by the OT team have a lower incidence of POD compared to the group treated only with standard measures.

**Methods:**

A multicenter, single-blind, randomized clinical trial was conducted between October 2018 and April 2021, in Santiago of Chile, at a university hospital and at a public hospital. Patients older than 75 years undergoing elective major surgery were eligible for the trial inclusion. Patients with cognitive impairment, severe communication disorder and cultural language limitation, delirium at admission or before surgery, and enrolled in another study were excluded. The intervention consisted of OT therapy twice a day plus standard internationally recommended non-pharmacological prevention intervention during 5 days after surgery. Our primary outcome was development of delirium and postoperative subsyndromal delirium.

**Results:**

In total 160 patients were studied. In the interventional group, treated with an intensive prevention by OT, nine patients (12.9%) developed delirium after surgery and in the control group four patients (5.5%) [*p* = 0.125, RR 2.34 CI 95 (0.75–7.27)]. Whereas subsyndromal POD was present in 38 patients in the control group (52.1%) and in 34 (48.6%) in the intervention group [*p* = 0.4, RR 0.93 CI95 (0.67–1.29)]. A *post hoc* analysis determined that the patient’s comorbidity and cognitive status prior to hospitalization were the main risk factors to develop delirium after surgery.

**Discussion:**

Patients undergoing intervention by the OT team did not have a lower incidence of POD compared to the group treated only with standard non-pharmacological measures in adults older than 75 years who went for major surgery.

**Clinical trial registration:**

www.ClinicalTrials.gov, identifier NCT03704090.

## 1. Introduction

Approximately 310 million surgeries are performed worldwide per year and more than 25% of them are accomplished on patients over 65 years old (1, 2). Depending on the complexity of the surgery and the comorbidities of the patients, up to 50% of older adult patients may develop postoperative delirium (POD) (1, 2). Patients who develop POD have worse outcomes, such as increased morbidity, increased hospital stay, higher hospital costs, cognitive and functional impairment, and higher mortality (1–4). In addition, it has been determined that delirium can be prevented and, therefore, effective measures of prevention should be established to generate benefits in the health of patients and reduce health costs (5).

In order to reduce delirium’s incidence, non-pharmacological prevention measures must be implemented (5, 6). The aforementioned measures includes, prior to surgery, the use of precipitating drugs should be avoided, fasting times should be reduced, the sleep-wake cycle maintained, and any predisposing factors of delirium should be recorded (7, 8). Meanwhile, intraoperatively, complications should be diagnosed and treated early on. Benzodiazepines and atropine among other drugs should be avoided, nociception must be adequately managed, and the depth of the anesthetic must be monitored (7–9). Finally, during the postoperative period, guidance and environmental management measures must be established (5, 7, 10). However, the prevention of POD is still ineffective in the perioperative period and therefore, it is necessary to continue exploring feasible protocols to prevent POD. It has been documented that intervention with occupational therapy (OT) teams reduces the incidence and duration of delirium compared to a standard intervention in critically ill medical patients (11). That is, in patients who did not undergo surgery, but were admitted to a critical patient unit due to a medical pathology, such as pneumonia, OT therapy prevented the occurrence of delirium from 20 to 3% of patients.

For this reason, we designed a prospective clinical study with the aim to determine whether patients undergoing intervention by the OT team have a lower incidence of POD compared to the group treated only with standard measures, in a group of adults over 75 years old who underwent major surgery. Our hypothesis is that non-pharmacological prevention of POD performed by OT teams will decrease the incidence rate of delirium compared to standard prevention therapy in patients over 75 years old undergoing highly complex elective surgeries.

## 2. Materials and methods

### 2.1. Methodological design

This is a prospective, single-blind, parallel-group, randomized clinical trial conducted in two Chilean hospitals [Hospital Clínico de la Universidad de Chile (HCUCH) and Complejo Hospitalario San José (CHSJ)]. Recruitment occurred between October 2018 and April 2021. This study was approved by the University’s Institutional Review Board (IRB OAIC N° 926/17, November 2017) and written informed consent was obtained from all subjects participating in the trial. The trial was registered prior to patient enrollment at clinicaltrials.gov (NCT03704090). Details of the original study protocol are available in Supplementary material 1. In addition, the study was designed following CONSORT recommendations (12) for reporting non-pharmacological trials. Regarding the design of the protocol, the TIDieR (13) and SPIRIT standards were used (14). In this study, patients older than 75 years old, who were scheduled for major elective non-cardiac surgery (Supplementary material 2), in one of the two centers, were invited to participate prior to surgery and were asked to sign the informed consent. After that, the assessments and the corresponding interventions were carried out until the 5th postoperative day or until discharge, depending on which occurred first. During the recruitment period, the study was affected by the COVID-19 pandemic. For this reason, the recruitment was suspended between April and October 2020. Subsequently, the sample was completed with the last 25 patients. The only difference from the previous period was that family visits were suspended.

### 2.2. Study population

Patients with the following characteristics were excluded: cognitive impairment prior to admission [Mini-Mental State Examination (MMSE) with < 23 points in the case of 6 or more years of schooling and < 18 points in the case of < 6 years of schooling] (15); severe communication disorder and cultural limitation due to language (language other than Spanish); delirium upon admission or prior to the start of surgery; and enrollment in another study. V.R., V.L., and R.G. enrolled the patient, and the randomization was performed by E.T. Enrolled patients were randomized with a computerized system of code assignment, with a simple randomization to the control group or intervention group and a stratification with a 1:1 ratio in blocks of 10 subjects per center. Randomization was sequentially numbered with the use of sealed and opaque envelopes and was reported to the coordinator of each center.

### 2.3. Study interventions

A standard non-pharmacological delirium prevention system was applied to the control and interventional groups in the postoperative period (Supplementary material 3). The standard measures included: reorientation protocol, performed by the nursing team, consisting of directly informing the patient at least 3 times a day of the time, date, place, and reason for hospitalization; early mobilization, performed by a physiotherapy team twice a day; sensory deficit correction, it encourages the use of correctors and technical aids such as glasses, hearing aids, and dentures, among others; environmental management, installation of a clock and other orientation elements in the patient’s room to promote orientation, in addition to minimizing environmental stressors; sleep protocol, lowering of lights, noise, and administration of nighttime drugs; hydration protocol, monitoring of the patient’s hydration and access to it; and reduction of medication.

Interventional group also received an additional non-pharmacological prevention intervention carried out by an OT team (Supplementary material 4). This intervention consisted of two daily personalized stimulation sessions of 25 min each, separated by an interval of at least 4 h. During each session, the following was carried out: cognitive stimulation, intervention aimed at keeping mental functions active; polysensory stimulation, providing the patient with intense external stimulation regulated by different sensory channels cognitive and motor stimulation of the upper extremities; positioning, providing early installation of orthosis and adaptations that leave areas with the highest frequency of bedsores free of pressure; basic training in Activities of Daily Living (ADL), the intervention will focus on encouraging ADL such as hygiene, grooming and nutrition performed independently; motor stimulation of upper limbs, it consisted of maintaining or activating functional movements and the strength of the upper extremities; and participation of family, consisted of the incorporation of the family in the health interventions.

### 2.4. Study procedures

After patient consent, MMSE, Functional Independence Measure (FIM) (16), functional comorbidity index, Charlson comorbidity index (CCI) and confusion assessment method (CAM) (17) were performed to the patients. In addition, general demographic and clinical data were recorded. Then, the corresponding surgery was performed according to the internal protocols of each institution. From the morning after surgery, the CAM was applied twice a day (morning and afternoon) for 5 days or until discharge (depending on which occurred first), to determine whether the patient developed delirium. The evaluators were occupational therapists who underwent CAM training to perform the questionnaire. On the 5th day or at discharge, the MMSE and FIM were applied again, and grip force measurement was performed for both upper extremities with a dynamometry (18). Finally, mortality was recorded 30 days after surgery. To coordinate each of the activities, one coordinator was assigned in each of the centers. They allowed the evaluation group to remain blinded and they did not participate in the data analysis, that was also done blindly.

### 2.5. Study outcomes

The primary outcomes were the incidence of POD determined with CAM during the first 5 days after surgery or until discharge (whichever occurred first); and the incidence of subsyndromal POD (PODS). PODS was defined as any alteration in the CAM that does not meet the diagnostic criteria for POD (19). In addition, the following secondary outcomes were compared: length of hospital stay; mortality, the number and percentage of patients who died 30 days after surgery were recorded; and duration of delirium, where the number of evaluations in which patients had delirium was measured.

### 2.6. Statistical analysis

The sample size was calculated based on the primary outcome of the incidence of POD. In a previous study of our team, it was found that 21% of patients > 75 years old presented POD during the first 5 days after surgery (20). It was estimated that the intervention by the group of occupational therapists could decrease the incidence of POD from 20 to 5%, since this decrease had been previously observed by our group in critical ill patients of non-surgical medical causes (11). Thus, considering a power of 80% and a two tailed alpha of 0.05 and considering a 10% loss in the follow-up of the patients, a sample size of 80 patients per group was calculated with http://powerandsamplesize.com/.

Data were analyzed with intention to treat. For descriptive analysis of the continuous variables with normal distribution, the mean (standard deviation) was used, the variables without normal distribution were used as median (25–75 percentiles) and the categorical qualitative variables were used as a percentage. The analysis of the primary outcome of development of POD and PODS was performed with Fisher’s exact test. The other analyzes were performed using the Student’s *t*-test, the Mann-Whitney-Wilcoxon test, Fischer’s exact test, and chi-square test as appropriate. Percentage of OT intervention were calculated as the number of OT interventions performed divided by all potential OT intervention. While percentage of CAM evaluations were the number of CAM evaluations performed divided by all potential CAM evaluations. An α of 0.05 was considered to reject the null hypothesis. Stata 17 and GraphPad Prism 9 were used for the analysis.

Subsequently, two *post hoc* analyses were performed. In the first, the patients were divided into three groups: POD, PODS, and no delirium; and then, a univariate analysis was performed using the Kruskall-Wallis test, one-way ANOVA, and the chi-square test according to the distribution of variables. In the second, a multivariate analysis was performed using a conditional classification tree, with the objective of exploring the impact of the studied variables on the negative outcomes of the patients (POD and PODS). The steps used to perform this analysis were: first, we start evaluating the composite scores of relevant variables for patients’ outcomes (Age, scholarity, CCI, functional comorbidity index, initial MMSE, initial cognitive, and motor FIM). Composite scores were developed using Exploratory Factor Analysis. To detect the number of dimensions (scores) to be extracted, we used parallel analysis (21, 22). Then, we extracted the dimensions using Principal Axes Factoring (23, 24). Finally, we estimated composite scores using the Thurstone method (regression-based weights). Those composite scores were used as predictors of POD using Conditional Classification Trees (25). This approach allowed us to evaluate potential contributions of variables of interest, extracting potential cutoffs to anticipate POD or PODS. Conditional Classification Trees analysis was performed with R Core Team (26).

## 3. Results

### 3.1. Characteristics of patients

**FIGURE 1:**
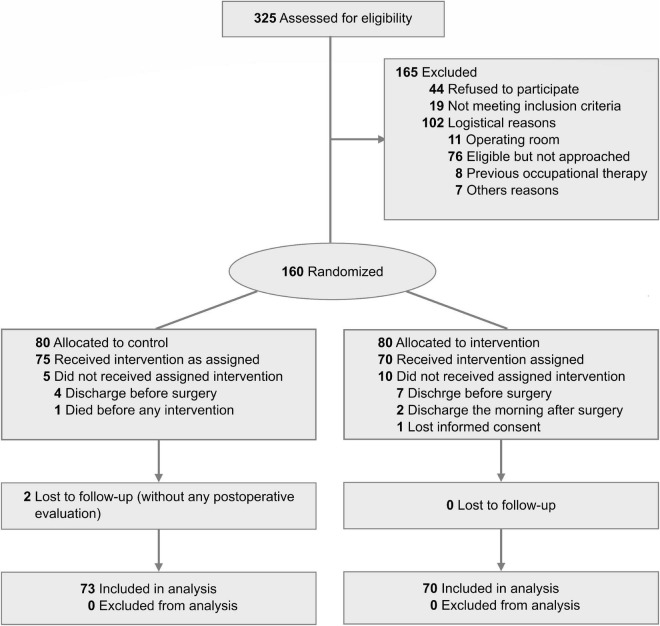
CONSORT diagram. Of the 325 patients eligible for screening, 160 patients provided consent and were randomized, 80 standard non-pharmacological delirium prevention of control group and 80 to occupational therapy intervention. Overall, 73 patients were analyzed for the control group and 70 for the intervention group, which concluded their interventions and evaluations.

A total of 160 patients were recruited, of which 17 (10.6%) had to be excluded: 11 patients did not undergo surgery, four did not receive postoperative follow-up, one patient had no record of informed consent, and one patient died without any evaluation. Of the remaining 143 patients, 90 (62.9%) patients were recruited in the HCUCH and 53 (37.1%) subjects in the CHSJ (Figure 1). The control group consisted of 73 (51%) patients and the intervention group of 70 (49%) patients, which were comparable to each other in the basal variables (Table 1). Finally, 332 OT interventions were carried out of the potential 452 in the intervention group (73.5%). Five (7.1%) subjects of this group could not undergo OT interventions, while the remaining received a median of 5 (3–7) interventions.

**TABLE 1:** Characteristics of patients at baseline.

Characteristic	Overall (*n* = 143)	Control (*n* = 73)	Intervention (*n* = 70)
Age, mean (SD)	78.97 (3.87)	79.2 (4.07)	78.7 (3.67)
Female, no. (%)	80 (56)	41 (56)	39 (56)
**Type of surgery, no. (%)**			
Orthopedic	76 (53)	42 (58)	34 (49)
Urologic	40 (28)	19 (26)	21 (30)
General	27 (19)	12 (16)	15 (21)
**Education level, no. (%)**			
≤6 year	47 (34)	24 (34)	23 (34)
7–12 years	67 (49)	35 (49)	32 (48)
≥13 year	24 (17)	12 (17)	12 (18)
CCI, median (IQR)	4.5 (3–6)	4 (3–6)	5 (4–6)
MMSE, median (IQR)	27 (25–29)	27 (25–28)	27 (25–29)
FIM motor, median (IQR)	89 (83–90)	88 (83–90)	89 (83–90)
FIM cognitive, median (IQR)	35 (34–35)	35 (34–35)	35 (34–35)

CCI, Charlson comorbidity index; IQR, interquartile range; MMSE, Mini-Mental State Examination; FIM, functional independence measure.

### 3.2. Primary outcome

In total 13 (9.1%) patients developed POD and 72 (50.4%) developed PODS. In the primary outcome no differences were found between both groups. The incidence of POD in the control group was 5.5% (4 patients) and in the intervention group was 12.9% (9 patients) [*p* = 0.125, RR 2.34 CI 95 (0.75–7.27)]. Whereas the incidence of PODS was in the control group 52.1% (38 patients) and in the intervention group was 48.6% (34 patients) [*p* = 0.4, RR 0.93 CI 95 (0.67–1.29)] (Table 2). Furthermore, there was no difference in the percentage of CAM evaluations between groups [control 443/469 (94.5%) vs. intervention 427/452 (94.5%), *p* > 0.99].

**TABLE 2:** Primary and secondary outcomes.

Outcome	Overall (*n* = 143)	Control (*n* = 73)	Intervention (*n* = 70)	*P*-value
**Primary outcome**				
POD, no. (%)	13 (9.1)	4 (5.5)	9 (12.9)	0.125
**Secondary outcomes**				
PODS, no. (%)	72 (50.3)	38 (52.1)	34 (48.6)	0.68
POD days, median (IQR)	1 (0.5–1.5)	0.75 (0.5–1)	1 (0.5–2.25)	0.46
PODS days, median (IQR)	1 (0.5–2)	1.25 (0.5–2)	1 (0.5–1.5)	0.36
Hospital LOS, median (IQR)	4 (3–6)	4 (3–6)	4 (3–6)	0.72
Mortality at 30 days	0	0	0	
Hand grip (right), median (IQR)	19 (15–30)	19 (14–30)	19 (15–30)	0.53
Hand grip (left), median (IQR)	19 (13–27)	19 (13–25)	19 (13–28)	0.79
MMSE, median (IQR)	27 (25–29)	27 (26–29)	27 (24–29)	0.57
FIM motor, median (IQR)	50 (34–61)	47 (34–61)	55 (37–62)	0.32
FIM cognitive, median (IQR)	34 (33–35)	34.5 (33–35)	34 (32–35)	0.93

POD, postoperative delirium; PODS, POD subsyndromal; IQR, interquartile range; LOS, length of stay; MMSE, Mini-Mental State Examination; FIM, functional independence measure.

### 3.3. Secondary outcomes

LOS, mortality at 30 days, duration of POD and PODS were similar in both groups (Table 2). Regarding other evaluated outcomes, we observed that there was no difference in independence, motor and cognitive FIM, handgrip and MMSE at discharge.

### 3.4. *Post hoc* analysis

In the univariate analysis was observed that the patients with POD were older, had a lower motor and cognitive FIM, and a lower MMSE at the time of recruitment (Table 3). While, in the postoperative period, LOS was longer in patients who developed delirium after surgery, and the patients who suffered POD had a greater decrease in MMSE, and motor and cognitive FIM than the other two groups (Table 3).

**TABLE 3:** *Post hoc* univariate analysis between POD, PODS and without delirium patients.

Characteristic	POD (*n* = 13)	PODS (*n* = 61)	Without delirium (*n* = 69)	*P*-value
Age, mean (SD)	82.6 (4.5)	78.5 (3.2)	78.7 (4.0)	0.002
Female, no. (%)	7 (54)	35 (57)	31 (45)	0.95
Type of surgery, no. (%)				0.37
Orthopedic	4 (31)	32 (52)	39 (57)	
Urologic	6 (46)	18 (30)	15 (22)	
General	3 (23)	11 (18)	15 (22)	
Education level, no. (%)				0.63
≤6 year	5 (39)	22 (37)	21 (31)	
7–12 years	6 (46)	30 (51)	31 (46)	
≥13 year	2 (15)	7 (12)	15 (22)	
CCI, median (IQR)	5 (4–7)	4 (3–6)	4 (3–5)	0.1
MMSE basal, median (IQR)	25 (23.5–27.5)	25 (27–28)	28 (26–29)	0.002
MMSE final, median (IQR)	22 (21–25)	26 (24–28)	28 (27–29)	<0.0001
MMSE change, median (IQR)	2 (0–5)	0 [(–1)–2]	0 [(–1.75)–1]	0.03
FIM motor basal, median (IQR)	82 (64.5–89)	89 (84–90)	89 (85–90)	0.026
FIM motor final, median (IQR)	23 (16.5–33.75)	55.5 (41–62.75)	51 (40–61.25)	<0.0001
FIM motor change, median (IQR)	48 (35.75-68.5)	32.5 (22-45)	35 (25.5–45.75)	0.01
FIM cognitive basal, median (IQR)	35 (30.5–35)	35 (34–35)	35 (35–35)	0.037
FIM cognitive final, median (IQR)	27.5 (17–30.75)	34 (33–35)	35 (33–35)	<0.0001
FIM cognitive change, median (IQR)	6.5 (2–12)	0 (0–2)	0 (0–1)	<0.0001
Hand grip (right), median (IQR)	15 (12–25)	18 (14–30)	21 (15–31)	0.1
Hand grip (left), median (IQR)	16 (8–21)	17 (12–27)	20 (15–27)	0.06
Hospital LOS, median (IQR)	6 (4.5–16)	5 (3.5–6.5)	4 (2.5–5)	0.002

POD, postoperative delirium; PODS, POD subsyndromal; CCI, Charlson comorbidity index; IQR, interquartile range; MMSE, Mini-Mental State Examination; FIM, functional independence measure; LOS, length of stay.

**FIGURE 2:**
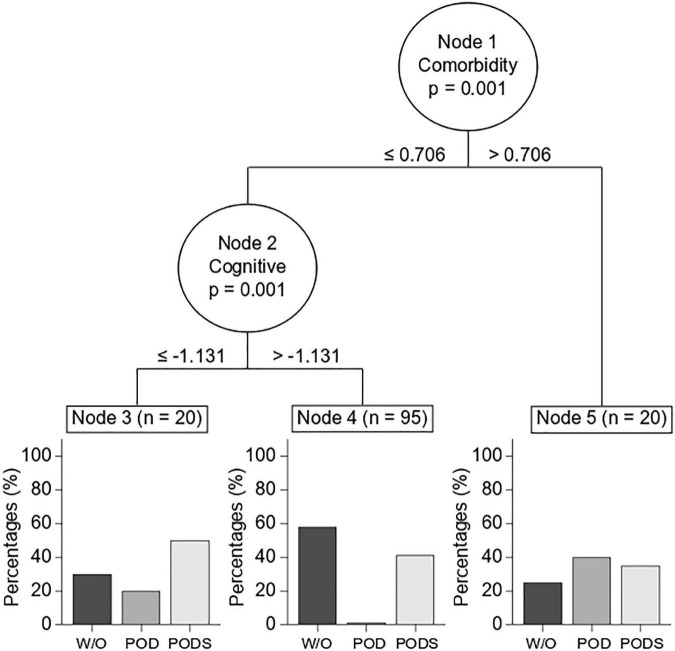
Conditional classification tree (*post hoc* analysis). To assess the ability to discriminate against patients who developed POD, variables were grouped into three different dimensions (comorbidity, cognition, and motor). The dimensions of comorbidity (Node 1) and cognition (Node 2) showed a significant level of discrimination against patients who developed POD. Nodes 3, 4, and 5 show the incidences of POD development in patients with an intermediate risk (node 3), low risk (node 4), and high risk (node 5) for developing POD.

On the other hand, in the classification tree it was observed that the dimension of comorbidities had hierarchically greater relevance than the dimension of cognitive status (Figure 2). Thus, patients with a composite comorbidity score greater than 0.706 had a delirium rate close to 40%. In subjects with a score lower than 0.706, the cognition area allowed to identify patients with a higher risk. Patients with a score greater than –1,131 in the cognition area practically did not develop delirium, while the POD rate was approximately 20% in those with a score lower than –1,131. Finally, the efficacy of the OT intervention was studied in the different subgroups determined by the classification tree. It was observed that the patients in the intervention group with a lower comorbidity score had a lower incidence of PODS (control 13/35 (37.1%) vs. intervention 3/26 (11.5%), *p* = 0.001) (Table 4).

**TABLE 4:** Developing POD or PODS into comorbidity-level groups by classification tree analysis.

Outcome	Group (classification tree)	Control	Intervention	*P*-value
POD, no. (%)	High comorbidity	3 (6)	7 (14)	0.18
	Low comorbidity	1 (4.3)	1 (4.2)	≈1
PODS, no. (%)	High comorbidity	18 (27.7)	21 (32.8)	0.395
	Low comorbidity	13 (37.1)	3 (11.5)	0.001

POD, postoperative delirium; PODS, POD subsyndromal.

## 4. Discussion

The intensive prevention of POD in elderly patients carried out by an OT team, after surgery, did not decrease the incidence of POD and PODS compared to the use of standard delirium prevention measures. However, the overall POD incidence of 9.1% is lower than that reported in patients older than 75 years undergoing more complex surgeries (27, 28). In general, it has been reported that patients aged 75 years or older are highly vulnerable to experiencing POD (3, 28).

One point of interest worth discussing is understanding why OT interventions did not elicit significant improvements compared to standard delirium prevention. Some possible causes could be investigated: (i) standard non-pharmacological prevention measures may have generated a maximum effect in terms of prevention capacity, rendering intensive OT measures ineffective (5, 10). In our study, we implemented standard recommended prevention measures, which have been reported to be effective for prevention (5). Perhaps in delirium after surgery these measures are enough to prevent episodes of POD; (ii) the baseline conditions of the patients, such as age and cognitive status, are more relevant for the developing of POD than the use of preventive strategies (1); (iii) intensive prevention measures could have been effective prior to surgery, whereas in our study they were implemented in the postoperative period (29–33). In a recent article, it was shown that prehabilitation through the stimulation of cognitive functions could be effective in preventing POD (34). In addition, it has been shown that prior to surgery, monitoring predisposing risk factors and previous cognitive conditions are relevant for prediction and in this way preventive measures could be focused (34–37). Perhaps by carrying out these more focused intensive prevention measures in predisposed patients, a significant effect could be found. However, interventions prior to surgery are often difficult to perform, while our study addressed interventions that are more feasible to perform. We consider that standard non-pharmacological prevention measures after surgery should be established in all patients at risk.

In the secondary outcomes, there was also no significant difference between the groups. The intensive intervention with OT during the postoperative period did not generate an improvement in any outcome that was evaluated. In the group that manifested POD, 68.6% of the potential interventions were performed, while in those that did not manifest delirium, 76.4% of the potential interventions were performed. This difference was not significant, but patients who developed POD had a lower number of interventions performed, which can be explained by the fact that a patient with delirium is more difficult to receive OT intervention. On the other hand, the low incidence of delirium in our sample may be an indicator that there was a low rate of complications in the postoperative period and, consequently, it could explain the low rate of mortality at 30 days (35, 36). However, the CCI in our cohort of patients was relatively high. It may be that the high rate of orthopedic surgeries (53%) and the early discharge protocols used in this type of surgery, in our study, could explain the low morbidity and mortality after surgery.

Patients who developed delirium were older, with lower cognitive performance and less independence in ADL at hospital admission, which has been widely reported (19, 37, 38). Interestingly, in our analysis of the conditional classification tree it was determined that POD was hierarchically predisposed first by comorbidities and then by basal cognitive status. In the current literature, the initial cognitive status of patients has been determined as a predisposing factor for developing POD in various hospital units (3). For this reason, it is advisable to evaluate the preoperative cognitive reserve in older adult patients with a cognitive evaluation (39, 40). About comorbidities, a systematic review and meta-analysis identified that the combination of comorbidity and delirium was present in 54% (95% CI: 39–69%, 20 studies and 1,346 participants) (36). What is interesting about the analysis presented here is that the discriminatory capacity of these factors is hierarchized, which might allow to select patients to intervene intensively with prevention measures.

In our study, patients without delirium had a shorter hospital stay compared to other reports (28, 41). Most likely, the early discharge protocols for major trauma surgery, which are used in both centers of our study, would explain this difference. Furthermore, the patients who developed POD had a greater decrease in motor FIM and cognitive FIM. This finding is related to reports that the development of POD delays the recovery of patients after surgery (42).

A relevant aspect for OT intervention is to improve the independence of the subjects during their hospital period, which can be objectified with the FIM. In the case of our sample, no difference was observed before surgery between groups. Regarding the effect of the intervention, it was observed that in patients who received OT therapies, the motor FIM fell from 89 to 55 points, which is explained by the surgical intervention. Meanwhile, a similar decrease occurred in the control group, falling from 88 to 47 points. This indicates that there was no demonstrable effect of the OT intervention. In contrast, in a study previously published by our group, it was observed that in non-ventilated elderly patients in a critical care unit, patients undergoing OT intervention increased their motor FIM significantly more than the control group (11). This indicates that in the context of the medical patient, the intervention did have a verifiable effect. On the other hand, the patients who developed POD had a greater decrease in motor FIM than the patients who did not have delirium or who had PODS. Therefore, patients with POD could benefit from continued OT care after discharge.

Our study has several limitations. First, the incidence of delirium was lower than expected. A 20% incidence of POD was expected, but only 9.1% of the patients developed POD. This makes the capacity of the study to find differences lower than expected. However, most likely, this was not a problem because the intervention group had a higher incidence of POD (12.9 vs. 5.5%), which indicates that the intervention does not prevent POD. Furthermore, the low POD rate observed cannot be attributed to the lack of detection, since 94.5% of the possible evaluations with the CAM were performed. Second, the intervention was carried out after the surgical injury. Given the findings of cognitive pre-habilitation (34), the intervention should possibly begin preoperatively to increase effectiveness. This could be part of a future study. Third, the study was designed in a group of high-risk patients, by age and by type of surgery, and it could very well be that prevention in this group is less feasible. In fact, in our *post hoc* analysis, it was preliminarily observed that patients with a lower comorbidity score had a lower rate of PODS when undergoing OT. This would indicate the opposite of what one could intuitively hypothesize; thus, preventive measures could be more effective in patients with a lower risk and less effective in those with a higher risk. Fourth, the focus of our study was to record pre- and postoperative data. Unfortunately, in the intraoperative period we only recorded the type of surgery, and we did not do so for the type of anesthesia or the surgical time, which would have been desirable to show. Finally, we did not measure inter-rater and intra-rater reliability before or after the study.

## 5. Conclusion

Post-operative OT intervention was not superior to standard prevention in decreasing the presence of POD and PODS in adults older than 75 years old. However, the incidence of POD was low in both groups, compared to previous studies. On the other hand, independence, comorbidity, and cognitive status factors are factors that contribute to the presence of POD and PODS.

## Data availability statement

The raw data supporting the conclusions of this article will be made available by the authors, without undue reservation.

## Ethics statement

The studies involving human participants were reviewed and approved by the University’s Institutional Review Board (IRB OAIC No. 926/17, November 2017), Hospital Clínico de la Universidad de Chile. The patients/participants provided their written informed consent to participate in this study.

## Author contributions

EA, DP, FS, ET, CB, and AP contributed to the design of the research. VR, LC, MG, DP, CB, and AP were worked the implementation. RG, FR, RV, JE, and AP contributed to the analysis of the results. All authors contributed to the article and approved the submitted version.
