# Supplementary material for: Non-pharmacological prevention of postoperative delirium by occupational therapy teams: A randomized clinical trial
Source: Front Med (Lausanne). 2023 Feb 2;10:1099594. doi: 10.3389/fmed.2023.1099594 (PMC9931896; doi:10.3389/fmed.2023.1099594)
Supplement: Supplementary file 1 [file Table_1.docx]

**Supplement 1**

**Research Protocol**

**Table of contents**

| **1.** Title and Summary | Page 2 |
| --- | --- |
| **2.** Introduction | Page 3 |
| **3.** Methods and Procedures | Page 6 |
| **4.** Interventions | Page 8 |
| **5.** Outcomes | Page 13 |
| **6.** Sample Size | Page 15 |
| **7.** Interim analysis | Page 16 |
| **8.** Randomization | Page 17 |
| **9.** Allocation concealment mechanism | Page 18 |
| **10.** Implementation | Page 19 |
| **11.** Blinding | Page 20 |
| **12.** Statistical methods | Page 21 |
| **13.** Other information | Page 22 |
| **14.** References | Page 23 |

**1a TITLE**

**Non-pharmacological Prevention of Postoperative Delirium by Occupational Therapy Teams: a randomized clinical trial**

**1b SUMMARY**

**Background:** It has been described that a quarter of all surgeries are performed on older adults, and that up to 50% of elderly patients may develop postoperative delirium (POD).[(1,2)](https://paperpile.com/c/YGPhmB/MaBdk+QfL5C) Patients with POD have worse short- and long-term clinical outcomes, such as increased postoperative morbidity, increased hospital stay, higher hospital costs, cognitive and functional impairment, and higher post-surgery mortality.^1–4^ In addition, it has been determined that delirium is preventable,^5^ and therefore, effective prevention measures should be established to generate benefits in the health of patients and reduce health costs. On the other hand, it has been seen that intervention with occupational therapy (OT) in non-mechanically ventilated elderly patients hospitalized in critically ill patient units, managed to significantly reduce the incidence and duration of delirium compared to a standard intervention.^6^ However, we do not know whether OT interventions can be effective in this population.

**General Objective**: To determine the efficacy of a non-pharmacological preventive intervention performed by occupational therapy teams in the incidence of postoperative delirium (POD) and POD subsyndromal (PODS) in patients older than 75 years undergoing highly complex elective surgeries.

**Design/Method:** Randomized clinical trial (RCT), multicenter, prospective, with parallel groups, in a 1:1 ratio, in 2 Chilean hospitals (Hospital Clínico Universidad de Chile (HCUCH) and in the Complejo Hospitalario San José (CHSJ)).

**Participants:** Patients aged 75 years or older, admitted to the hospital, who will undergo highly complex elective surgery at the HCUCH or CHSJ, and who have provided their consent, will be studied. Patients with known cognitive impairment prior to admission, severe communication disorder and cultural limitation of language, delirium upon admission or prior to the start of the intervention, and patients enrolled in another study will be excluded.

**Intervention:** A control group (standard non-pharmacological prevention of delirium measures) or an intervention group (early and intensive OT + standard non-pharmacological prevention of delirium measures) will be randomized. The intervention group will receive 2 daily sessions of 30 minutes, for 5 days, receiving a total of 10 OT sessions, which considers a predefined protocol of actions according to the patient's condition.

**Evaluations:** The presence of POD and PODS will be evaluated with the Confusion Assessment Method (CAM), mortality, functional independence, and motor and cognitive state at discharge. In addition, sociodemographic and clinical data will be included.

**2a INTRODUCTION**

Around the world, 230 million surgeries are performed per year and a third of the patients who undergo surgeries are over 65 years old.[(3,4)](https://paperpile.com/c/YGPhmB/wYG4j+a74uw) Worryingly, depending on the complexity of the surgery, between 10 and 70% of elderly patients develop POD.[(1,2)](https://paperpile.com/c/YGPhmB/MaBdk+QfL5C) These patients with POD have poor short- and long-term clinical outcomes, such as an increase in postoperative morbidity, a prolonged hospital stay, a deterioration in functional and cognitive status, and even an increase in mortality one year after surgery.[(1,2,5,6)](https://paperpile.com/c/YGPhmB/MaBdk+QfL5C+UrXRC+ipt2K) Consequently, these poor results are associated with a poorer quality of life of the elderly and an increase in health costs. Delirium has been determined to be preventable through various non-pharmacological interventions,[(7,8)](https://paperpile.com/c/YGPhmB/XLxQg+FZcGe) which constitute the first-line preventive tools, and which have the best clinical results. Consequently, they reduce the costs of health care for the elderly. However, both prevention and diagnosis of POD are still deficient in routine clinical practice worldwide and in our country. Therefore, it is essential to create protocols and recommendations that not only allow the prevention of POD but are also applicable in the health centers that serve the elderly adults who will undergo various surgeries.

POD is the delirium that occurs after surgery and is defined by the DSM-5 as an acute and fluctuating disorder, characterized by an altered state of consciousness, loss of attention and disorganized thinking.[(9)](https://paperpile.com/c/YGPhmB/9XO7q) This condition occurs during the first week after surgery and lasts an average of 3 days.

***POD Risk factors and triggers***

It is accepted that POD is the result of the interaction of risk factors and triggering factors, which ultimately alter brain function, causing acute cognitive impairment.[(10,11)](https://paperpile.com/c/YGPhmB/Bws4y+aUNwV) The risk factors of patients associated with POD are: older age, less schooling, malnutrition, a higher rate of comorbidity, a history of alcohol abuse and having a cognitive alteration prior to surgery.[(6,12)](https://paperpile.com/c/YGPhmB/ipt2K+EBNPA) Regarding surgical injury, if it is on a larger scale, the possibility of developing POD increases since the systemic inflammatory response is more intense and blood losses are more abundant, causing a decrease in cerebral perfusion.[(11)](https://paperpile.com/c/YGPhmB/aUNwV) The types of surgeries that have a greater association with POD are cardiac surgeries (46-70%),[(5,13)](https://paperpile.com/c/YGPhmB/UrXRC+OsXIE) major abdominal surgeries (18-24%)[(14,15)](https://paperpile.com/c/YGPhmB/v0D4a+uVprT) and hip replacement surgeries (3.6-53-3%).[(4,16)](https://paperpile.com/c/YGPhmB/a74uw+RgLNq) Finally, some drugs used in anesthesia increase the risk of developing POD, such as anticholinergics (atropine), benzodiazepines, opioid derivatives (morphine, fentanyl and tramadol),[(10,17)](https://paperpile.com/c/YGPhmB/Bws4y+IUAbX) in addition to the hypnotic depth evaluated by BIS,[(18)](https://paperpile.com/c/YGPhmB/z1x6g) among others. In short, these factors work together to alter brain function, which underlies the development of POD.

Therefore, when faced with a risk patient who will undergo major surgery, preventive measures can be taken to reduce the development of POD, such as avoiding the use of certain drugs, maintaining adequate cerebral perfusion, and reducing systemic inflammation.

***Prevention of POD***

As mentioned at the beginning, the cost-benefit balance is higher with prevention measures than with therapeutic measures. Therefore, the focus of this proposal is the prevention of POD to implement the results in different public and private health centers in our country. Next, we will detail the recommended measures to prevent POD.^20^

- **Preoperative:** The use of certain drugs, such as benzodiazepines, anticholinergics should be avoided, decrease the fast times, maintain the circadian day-night rhythm and the use of sedative drugs such as α2-agonists (dexmedetomidine or clonidine) should be considered.[(17,19)](https://paperpile.com/c/YGPhmB/Ty2Dk+IUAbX) In addition, the risk of each patient must be adequately stratified in relation to the presence of predisposing factors and the surgery to which they will undergo.
- **Intraoperative:** At this stage, the approach is to diagnose and treat any complications that may occur early. In addition, the use of benzodiazepines and atropine in anesthetic management should be avoided, and nociception during surgery should be properly managed using techniques that reduce the use of opioid derivatives such as the use of regional anesthesia techniques. Finally, it is recommended to monitor the anesthetic depth to avoid very deep anesthesia.[(19,20)](https://paperpile.com/c/YGPhmB/Ty2Dk+TkOzl)
- **Postoperative:** After surgery, all patients should undergo non-pharmacological measures to prevent POD. These measures consist of stimulating patient orientation with the use of watches and communication; facilitate the early use of glasses and hearing aids; reduce ambient noise and facilitate patients' sleep; avoid the use of unnecessary intravenous and urinary catheters; stimulate mobilization and early feeding; and use multimodal analgesic techniques in order to reduce opioid doses.[(8,19)](https://paperpile.com/c/YGPhmB/FZcGe+Ty2Dk) In conjunction with the above, the POD should be investigated early, even from the post-anesthesia recovery unit.[(19)](https://paperpile.com/c/YGPhmB/Ty2Dk)

Regardless of the perioperative stage in which the prevention of POD is carried out, measures with a non-pharmacological approach and others that require the use of drugs can be described. It is noteworthy that non-pharmacological measures are less expensive, are more applicable in health systems, and there is evidence that they are effective in reducing POD rates.[(8,21)](https://paperpile.com/c/YGPhmB/FZcGe+p4TfB) In our vision as a group, which is supported by the literature, we believe that the measures with more evidence and a better cost-effectiveness profile are non-pharmacological preventive measures. For this reason, this proposal focuses on this type of measures covering the perioperative.

***Previous work of the research team on prevention of delirium.***

Part of the research team studied whether the intervention by OT prevents the development of delirium in elderly adults hospitalized in critical patient units.[(22)](https://paperpile.com/c/YGPhmB/b8oXl) This study was carried out at the HCUCH and enrolled a total of 140 hospitalized patients in the Critical Patient Unit without the need for mechanical ventilation. They were subdivided into 2 groups, the control group underwent standard non-pharmacological prevention measures for delirium, that is: time and space orientation measures, stimulation of early mobilization, correction of sensory deficits, establishment of a more welcoming environment with caregivers avoiding the use of physical limiting measures, sleep protection and avoiding the use of delirium-promoting drugs. The experimental group underwent the same standard protocol plus early and intensive OT, which consisted of polysensory stimulation, improving the positioning of the patient in bed, cognitive stimulation, motivation in performing the basic activities of daily life, stimulation of the activity of the upper extremities and fostering family participation. Interestingly, the rate of delirium decreased significantly from 20% in the control group to 3% in the treatment group, which in turn led to a decrease in the days with delirium and patients had better functionality at discharge. These results indicate that the measures carried out by OT are highly effective and suggest that in the perioperative context they could be very effective in preventing the development of POD in elderly patients.[(22)](https://paperpile.com/c/YGPhmB/b8oXl)

***Final considerations***

Every day, in all the hospital centers of the country, elderly adults undergo all kinds of surgeries and what is required is that they regain their functionality as soon as possible. The multidisciplinary team in this scenario is critical and rehabilitation must be early and intensive, on a functional and cognitive level since these are the critical elements for an adequate recovery. Our research group has determined that the interventional work of OT is essential in the prevention of cognitive impairment in patients in the Critical Patient Unit. Therefore, it is relevant and necessary to study whether this therapy is effective in the perioperative period to prevent the development of POD.

**2b HYPOTHESIS AND OBJECTIVES**

The present study hypothesizes that the non-pharmacological prevention of postoperative delirium carried out by occupational therapy teams decreases the incidence rate of delirium compared to standard prevention therapy in patients older than 75 years of age undergoing highly complex elective surgeries.

The primary objective of the study is to determine the efficacy of a non-pharmacological preventive intervention performed by occupational therapy teams in the incidence of postoperative delirium and subsyndromal postoperative delirium in patients over 75 years of age who underwent highly complex elective surgeries.

Specific objectives include:

- To characterize adults over 75 years of age who underwent highly complex elective surgeries during the first 5 postoperative days.
- To evaluate the impact of non-pharmacological preventive intervention carried out by occupational therapy teams or standard prevention on the duration of delirium and hospital stay in patients over 75 years of age subjected to highly complex elective surgeries.
- To evaluate the impact of non-pharmacological prevention on the functionality of patients over 75 years of age undergoing highly complex elective surgeries.

**3 METHODS AND PROCEDURES**

**3a TRIAL DESIGN**

A randomized clinical trial (RCT) is proposed with a multicenter prospective, with parallel groups, in a 1:1 ratio, in 2 Chilean hospitals. The records will be made at the beginning of the study, during the interventions, and at hospital discharge.

For RCT planning, the CONSORT[(23)](https://paperpile.com/c/YGPhmB/4F2IU) methodological recommendations for reporting non-pharmacological trials are used, in addition to the TIDieR[(24)](https://paperpile.com/c/YGPhmB/ZcWDA) and SPIRIT standards.[(25)](https://paperpile.com/c/YGPhmB/FtlaA)

**3b PARTICIPANTS**

Patients older than 75 years of age, who must have a highly complex elective surgery at the HCUCH or CHSJ. The criteria are described in Table 1

**Table 1. Admission criteria**

| **Inclusion Criteria** | **Exclusion Criteria** |
| --- | --- |
| - Age equal to or greater than 75 years - Hospital admission for highly complex elective surgery - Consent by patient | - Known cognitive impairment prior to admission. Mini-Mental State Examination (MMSE) with <23 points in case the subject has 6 or more years of schooling and <18 points in case the subject has <6 years of schooling. - Severe communication disorder and cultural limitation of language (language other than Spanish) - Delirium at admission or prior to the start of the intervention (measured with CAM) - Patient enrolled in another study |

**3c CHARACTERISTICS OF CENTERS**

The research is carried out in 2 hospital centers in Chile. These are HCUCH and CHSJ. The first corresponds to a private institution and the latter to a public institution. Patients hospitalized in the surgery, urology, and trauma wards of the HCUCH and the urology and trauma wards of the CHSJ will be included. The units serve people over 18 years of age, who require admission due to the evaluation of advanced studies, medical stabilization, and surgical interventions.

All centers have specialists, doctors, nurses, physical therapists, occupational therapists, and nursing technicians. The Non-Pharmacological Prevention of Delirium protocol will be implemented in all the selected wards.

**3d CHARACTERISTICS OF CARE PROVIDERS**

Each center has a specific coordinator and executing staff. All standard and experimental care providers will undergo training on "Delirium and Non-Pharmacological Prevention Measures”, and they will oversee the multidisciplinary research team (anesthetists, geriatrician, nurse, physical therapist, occupational therapists) which will be held 2 months before recruitment, to achieve a homogeneous standard treatment.

**4 INTERVENTIONS**

**4a PREPARATION OF LOCAL TEAMS TO IMPLEMENT A PROGRAM OF NON-PHARMACOLOGICAL PREVENTION OF DELIRIUM MEASURES IN THE CENTERS THAT WILL PARTICIPATE IN THE CLINICAL TRIAL.**

To carry out this design, a series of activities must be performed to ensure each of the key elements of the design. Preparatory activities for the process are described as follows:

- **Assess barriers and perceptions about the non-pharmacological prevention of POD:** in each of the 2 centers, the work team will carry out an initial evaluation of the barriers and perceptions about the non-pharmacological prevention of POD and, in this way, the next step will be planned, which is the training of the different clinical services of the centers that will care for the patients in our study. For this, we will use a questionnaire that has previously used and validated.
- **Standard non-drug prevention training for POD:** the clinical services that will receive the patients recruited in this study will be trained to implement the standard non-pharmacological preventive measures by the work team of this proposal. This training will allow patients in both groups to apply these delirium preventive measures, after which the effect of the preventive intervention performed by occupational therapists can be assessed.
- **Train the recruitment, intervention, and evaluation teams:** the work team will train each member of the study to carry out an activity. The study will have a team of health professionals for the recruitment and registration of clinical data; with a team of occupational therapists who will perform the non-pharmacological prevention intervention; and a team of healthcare professionals for evaluations that will include the diagnosis of delirium and the completion of the questionnaire to assess functionality.

**Study groups**

**Control group - Non-Pharmacological Prevention of Delirium:**

Standard non-pharmacological prevention of delirium: non-pharmacological strategies are the first-line approach in the prevention of delirium and this type of prevention is recommended by experts in hospitalized elderly adults. Currently, studies on the subject support education and training as an important prevention strategy. The following measures were implemented for our protocol (see Table 2):

1. **Reorientation protocol:** performed by the nursing team, consisting of directly informing the patient at least 3 times a day of the time, date, place, and reason for hospitalization.
2. **Early mobilization:** performed by a physical therapy team twice a day. It includes a sequence of passive and active mobilization of limbs, rolling and supine activation, supine to sitting transference, sitting, sitting to standing transference, walking in place, and ambulation.
3. **Sensory deficit correction**, it encourages the use of correctors and technical aids such as glasses, hearing aids, and dentures, among others. For this, the nursing team will request the necessary implements from the family upon admission to the hospital and the patient will be reminded daily of their use.
4. **Environmental management:** installation of a clock and other orientation elements in the patient´s room to promote orientation, in addition to minimizing environmental stressors.
5. **Sleep protocol:** lowering of lights, noise, and administration of nighttime drugs.
6. **Hydration protocol:** monitoring of the patient's hydration and access to it.
7. **Reduction of medication:** mainly anticholinergics and minimization of the use of benzodiazepines.

**Table 2. Summary of standard delirium prevention protocol**

| **Action** | **Procedure** | **Responsible** | **Frequency** | **Materials** | **Method of delivery** | **Record** |
| --- | --- | --- | --- | --- | --- | --- |
| **1. Reorientation protocol** | Directly inform the patient of the time, day, place, and reason for hospitalization. | Team of nurses | 3 times a day | Verbalization of team | Direct contact with the patient | It will be monitored by a team, on paper or using an intranet registration system |
| **2. Early mobilization** | Perform motor and respiratory exercises. | Physical therapy team | 2 times a day | Resistance Band | Direct contact with the patient, which will gradually be eased up according to the physical and medical conditions. |  |
| **3.Correction of sensory deficits** | Encourage the use of correctors and technical aids (glasses, hearing aids, dentures). | Team of nurses, family | 1 time per day | Diptych with material | Direct contact with the patient and family |  |
| **4. Management of environment** | Install clock and other orientation elements in the patient´s room. Minimize environmental stressors. Avoid using physical restraints, replacing them with the company of family members or another member of the team. | Team of nurses, healthcare team who is treating the patient, family members | 1 time per day | Paper  Marker  Glue | Direct contact with the patient |  |
| **5. Sleep protocol** | Train the team in reducing lights, noise and administration of drugs.  Promote night rest and daytime activation. | Team of nurses and family. | 1 time a day | Record sheet or clinical chart | Direct contact with the patient |  |
| **6. Hydration protocol** | Monitor patient hydration and check indications / availability. | Team of nurses | 2 times a day | Record sheet.  Clinical chart | Direct contact with the patient |  |
| **7. Decrease of medication** | Manage polypharmacy, decrease drugs with anticholinergic potential, and minimize the use of benzodiazepines. | Healthcare team who is treating the patient | 1 time a day | Record sheet.  Clinical chart | Review of clinical chart |  |

**Interventional group – Early and intensive Occupational Therapy**

Regarding OT intervention, specific training must also be carried out for the group of occupational therapists, to properly implement the OTs´ own strategies.

The OT intervention is implemented for 5 consecutive days, 2 times a day, in sessions before noon, and sessions after noon, and each session lasts 30 minutes.

The interventions to be carried out are grouped into the following 6 prevention areas (Table 3):

1. **Polysensory stimulation**: consisting of providing the patient with intense external stimulation regulated by different sensory channels (visual, auditory, tactile, proprioceptive and gustatory), with the objectives of increasing alertness, preventing sensory deprivation and increasing interaction of the patient with the objects and people around him/her (GES-ACV 2013 Clinical Guide recommendation).[(26)](https://paperpile.com/c/YGPhmB/mZ0v7) Sensory stimulation protocols have proven to be effective in other pathologies, such as states of minimal consciousness and dementia.[(27)](https://paperpile.com/c/YGPhmB/zBbzD) States that share some elements with delirium, therefore, can be used in patients with POD.
2. **Positioning:** consists of the early installation of orthosis and adaptations that leave areas with the highest frequency of bedsores free of pressure (sacral region, heels, among others). Proper positioning of the patient makes it possible to reduce the appearance of joint stiffness, which generates a positive impact on the patient's functionality in activities of daily living and, therefore, a reduction in the burden for health providers and their family.[(28)](https://paperpile.com/c/YGPhmB/cxK4s) They also reduce the appearance of pressure ulcers, one of the conditions that causes longer hospital stays and higher costs, and finally, it allows to provide comfort to the patient during hospitalization.
3. **Cognitive stimulation**: intervention aimed at keeping mental functions active. According to the International Classification of Functionality these are: consciousness, orientation, attention, memory, calculation, praxis and language, which are included in 6 areas of action: i) attention and wakefulness, ii) visual perception, iii) memory, iv) calculation and problem solving, v) praxis and vi) language.[(22)](https://paperpile.com/c/YGPhmB/b8oXl)
4. **Basic Training in** **Activities of Daily Living (ADL)**: The intervention will focus on encouraging ADL such as hygiene, grooming and nutrition performed independently. There are many benefits of ADL training in hospitalized elderly adults. These allow to structure their time (generating daily routines that favor the maintenance of habits), maintain their independence (functional maintenance in the daily activities of the elderly person prevents and reduces their physical, psychological, and social deterioration) and promote the feeling of usefulness. Each patient must maintain a daily routine of activities of daily living with times for cleaning, hygiene, breakfast, lunch, tea, and dinner, in a normal environment (sitting on the side of the bed or out of bed). Independent performance will be encouraged, and the occupational therapist, nurse, nursing technician or family member will only be a guide for the safe execution of the activity.[(29,30)](https://paperpile.com/c/YGPhmB/lzCp2+rTuII) Information will be provided to family members with the necessary recommendations.
5. **Motor stimulation of upper limbs:** it consists of maintaining or activating functional movements and the strength of the upper extremities, through the performance of movements towards the midline with the use of objects, bimanual coordination, ergotherapies, prehensions, writing and use of therapeutic tools. The motor stimulation of upper extremities will be used as the necessary baseline for the optimal performance of the ADL, since it has been documented that the grip force is a good predictor of independence in the ADL, cognitive status and mortality in elderly people.[(31)](https://paperpile.com/c/YGPhmB/zWD7r)
6. **Participation of family:** the incorporation of the family in health interventions, particularly with the elderly, is a feasible and effective resource, as reported in some studies.[(32)](https://paperpile.com/c/YGPhmB/BTGi6) Therefore, their participation in this intervention is essential. It will begin with the identification of the family members who will collaborate with the intervention, who will be given information detailing their specific role. Likewise, they will be asked for material to work with, such as photographs or other significant elements for the elderly person.

**Table 3. Summary of occupational therapy protocol**

| **Action** | **Procedure** | **Responsible** | **Frequency** | **Materials** | **Delivery method** | **Record** |
| --- | --- | --- | --- | --- | --- | --- |
| **Polysensory stimulation** | Auditory, tactile, proprioceptive, vestibular, olfactory, and visual stimulation | Occupational therapist | Patient who is on level 1, 2 or 3, according to the SAS scale | Colognes, scents, brush, sponge, speakers, cream, flashlight | Direct contact with the patient | It will be monitored by an evaluator team, on paper or intranet registration system |
| **Positioning** | Evaluate according to conditions of movement and rest  Achieve a sitting position in bed, on the edge of the bed or armchair | Occupational therapy | 2 times a day | Polyurethane, cushions, armchair | Direct contact with the patient |  |
| **Cognitive stimulation** | Encourage the use of correctors and technical aids (glasses, hearing aids, dentures). | Occupational therapy | 1 time a day | Exercise manual, Paper  Marker | Direct contact with the patient and family |  |
| **Stimulation of upper limbs** | Install clock and other orientation elements in the patient´s room. Minimize environmental stressors. Avoid using physical restraints, replacing them with the company of family members or another member of the team. | Occupational therapist | 1 time a day | Exercise manual.  Water bottle. Resistance Band, Scissors, Paper, Pencil | Direct contact with the patient |  |
| **Training in Activities of Daily Living (ADL)** | Training in ADL, sitting on the edge of bed or in an armchair, feeding, modified bathing, transfers or changing room can be performed | Occupational therapist | 1 time a day | Comb, hairbrush, toothbrush, toothpaste, cologne, soap, towel, | Direct contact with the patient |  |
| **Family** | Delirium education and family stimulation | Occupational therapy | 2 times a week | Educational diptych | Direct contact with the patient |  |

The interventions will be noted in the patient's clinical chart and on the project sheet, to keep a record of the process, verify the implementation and adherence to the planned measures.

**5 OUTCOMES**

**Primary outcome**

Incidence of POD and PODS: Two evaluations will be performed per day with CAM for five consecutive days

**Secondary outcome**

- *DURATION OF POD AND PODS*. Two evaluations will be performed per day with CAM for five consecutive days.
- *MORTALITY.*
- *HOSPITAL STAY.*
- *FUNCTIONAL ASSESSMENT*, considers independence with FIM, cognitive status with MMSE, and grip strength with dynamometry.
- In addition, socio-demographic data, and clinical information (reason for admission, Charlson Comorbidity Index) will be collected.

**Assessment of primary outcome:**

INCIDENCE OF POSTOPERATIVE DELIRIUM (POD) AND POSTOPERATIVE SUBSYNDROMAL DELIRIUM (PODS). Confusion Assessment Method (CAM) was developed in 1988-1990, to improve the identification and recognition of delirium.^10^ CAM was intended to provide a new standardized method to enable non-psychiatrically trained clinicians to identify delirium quickly and accurately in both clinical and research settings. The CAM had a sensitivity of 94% (95% CI 91-97%) and specificity of 89% (95% CI 85-94%).[(33)](https://paperpile.com/c/YGPhmB/6osiQ) Generally, the entire CAM rating takes 5-10 minutes to complete.

Delirium incidence will be assessed using CAM, applied by a trained occupational therapist twice a day during the first 5 days of hospitalization including weekends. CAM positive diagnostic criteria is fulfilled by the presence of (1) Either acute change or fluctuation, obtained from a family member or nurse aware of the patient’s baseline mental status, and (2) Inattention assessed by difficulty focusing attention during conversation (being easily distractible or having difficulty keeping track of what was being said) or wrong attentional tests, plus either (3) Disorganized or incoherent thinking, defined as rambling or irrelevant conversation, unclear or illogical flow or of ideas, unpredictable switching from subject to subject during patient assessment, or (4) An altered level of consciousness defined as vigilant, lethargic, stupor or coma. The attentional tests used are month of the year or days of the week backwards, digit-span backwards (4 digits) or A test.

**Assessments of secondary outcomes:**

- DURATION OF DELIRIUM. CAM shall be used, with two evaluations per day for 5 consecutive days.
- MORTALITY. Review of clinical chart record and civil identification and registration service of Chile.
- HOSPITAL STAY. Review of clinical chart record
- FUNCTIONAL EVALUATION
  - *COGNITIVE STATUS.* The MMSE and the cognitive-behavioral scale to confirm and quantify the mental state, will be used. It is the most used guideline to measure this condition in studies of delirium in elderly people and it has been validated in Chile. It presents an alpha coefficient = 0.60 Inter-rater, and Kendall's correlation coefficient = 0.63. It is a tool used for detecting cognitive impairment. Its score ranges from 0 to 30. The levels of deterioration range from 24 to 30 points (without deterioration), from 18 to 24 (slight deterioration) and from 0 to 17 (severe deterioration).[(34)](https://paperpile.com/c/YGPhmB/qlaTr)
  - *INDEPENDENCE*. FIM will be used; it measures the functional level in 13 Activities of Daily Living (ADL), and 5 cognitive aspects for the performance of ADL, which has a scale of 1 to 7 (from less to greater independence). The scores range are between 18 and 126 points. It is performed by means of an interview with the patient, the direct caregivers, and through observation. This evaluation will be carried out at the time of discharge of the patients and at the 3-month follow-up. Functional independence is defined as the ability to score > 5 points in the specific domain of the FIM for 6 predefined ADLs (bathing, dressing, eating, grooming, transferring from bed to chair, and using the bathroom).[(35)](https://paperpile.com/c/YGPhmB/egezM)
  - MOTOR STATUS. The grip force will be evaluated using the Jamar Dynamometer which has a sensitivity of 86.7% and a specificity of 70.2%, with a Cronbach's alpha of 0.956-0.982 in the right upper limb and 0.956-0.984 in the left limb. The “American Association of Hand Therapist” protocol will be adapted for ICU patients.[(31)](https://paperpile.com/c/YGPhmB/zWD7r) This evaluation will be carried out at the discharge of the patients and at the third month of follow-up.

**6 SAMPLE SIZE**

It was calculated based on the primary outcome, which is the development of POD in the first 5 postoperative days or until discharge, whichever occurs first. In a previous study carried out at HCUCH, it was found that 21% of patients > 75 years of age presented POD in the first 5 postoperative days (unpublished data, presented at the Chilean Congress of Anesthesiology 2016). Furthermore, it was determined by our group that preventive therapy performed by occupational therapists decreased the incidence of delirium in patients hospitalized in the Critical Patient Unit of said hospital from 20% to 3%.[(22)](https://paperpile.com/c/YGPhmB/b8oXl) So, to detect an absolute decrease of 15% in the incidence of POD (from 20% in Group Control to 5% in Group Interventional), with a power of 80% and a two-tailed of 0.05, and considering a 10% loss in the follow-up of the patients, 80 patients per group should be studied. Therefore, 160 subjects must be recruited from patients > 75 years who underwent highly complex elective surgery.

**7 INTERIM ANALYSIS AND STOPPING INTERVENTIONS.**

A record will be kept of the interventions implemented in the patients i) standard non-pharmacological prevention of delirium measures to both groups, which will be observed by the evaluators and ii) OT intervention will be noted by the same care providers. These records will be evaluated by the responsible researchers.

In addition, analyzes will be carried out periodically to evaluate the recruitment rate, preventive therapies, data acquisition and possible problems not foreseen in the study design. These analyzes will be carried out every three months by the work team.

In these analyzes, lifting the blind or making projections of the results based on the recruited patients are not considered.

**8 RANDOMIZATION**

**8A METHOD**

The patients will be randomized using a computerized code assignment system and will be stratified in the 1:1 ratio.

**8B TYPE OF RANDOMIZATION**

The randomization system will be by separate blocks for each center (HCUCH and CHSJ), using a computerized code assignment system, with a simple randomization, in which there are 2 groups (control and interventional).

Patients will be assigned to a control or interventional group. All patients will have non-pharmacological delirium prevention interventions performed by doctors, physical therapists and nurses trained in a standard protocol. Patients in the interventional group will also receive sessions conducted by occupational therapists

**9 ALLOCATION CONCEALMENT MECHANISM**

This will be done by a researcher, who will carry out the registration and randomization process, saving the information on his/her personal computer.

It should be remembered that all patients, once recruited, will begin to receive care as per the standard non-pharmacological prevention of delirium measures measures.

**10 IMPLEMENTATION**

Recruitment will be carried out by one professional responsible for the health team of each center, who will identify and recruit potential patients. Once consent is obtained, they will inform the person in charge of randomization (independent statistician) via telephone message. The person in charge of randomization will notify the coordinator of each center which group the patient was assigned to, which may be a control or interventional group, starting the implementation of the study.

The coordinator of each center that carries out this process will not have contact with the evaluators, auditors, and the person responsible for the data analysis.

**11** **BLINDING**

In this study, the researchers, recruiters, evaluators, control group team and data analyst will be blinded. These teams will not have contact with the interveners of the interventional group. In the case of the evaluators who have contact with the patients of both groups, they will be professionals from other medical units, who do not know the team of interveners, and will only be able to structure their dialogue, according to the guideline of each evaluation.

It is not possible to mask the treating occupational therapist and patient receiving OT.

**12 STATISTICAL METHODS**

The data will be acquired both on paper and in an electronic database. However, to maintain the confidentiality of patients in the electronic database, only the recruitment code will be recorded and not the names. The recorded data will be analyzed by the researchers blind to the therapy. The primary outcome of this analysis will be to determine the incidence of POD and PODS at any time postoperatively, as previously defined. This analysis will allow us to establish whether a therapy performed by occupational therapists significantly reduces the incidence of POD. In addition, this analysis will have as secondary objectives to determine if the intervention by occupational therapists reduces the severity and duration of POD, and if it improves the functionality of the patients on the 5^th^ postoperative day.

**13 OTHER INFORMATION**

**Ethics Committee**

The research protocol was reviewed and approved by the ethics committee of the Hospital Clínico de la Universidad de Chile and its approval validated by the ethics committees of the respective participating centers (Registry No. 069 on November 2, 2017).

**Registry. Name and number of study registration.**

OAIC N°926/17

**Funding:** This project was funded by ‘Fondo Nacional de Investigación en Salud” (FONIS - SA17I0030), which belongs to the ‘Agencia Nacional de Investigación y Desarrollo’ (ANID). The commission did not participate in any stages of this study.

**Informed Consent**

Informed consent will be provided by a family member or patient and will be obtained by one of the main researchers.

**Declaration of interest**

The research team declares not to have conflict of interests.

**14 REFERENCES**

1. [Inouye SK, Westendorp RGJ, Saczynski JS. Delirium in elderly people. *Lancet* (2014) 383:911–922.](http://paperpile.com/b/YGPhmB/MaBdk)

2. [Nadelson MR, Sanders RD, Avidan MS. Perioperative cognitive trajectory in adults. *Br J Anaesth* (2014) 112:440–451.](http://paperpile.com/b/YGPhmB/QfL5C)

3. [Weiser TG, Regenbogen SE, Thompson KD, Haynes AB, Lipsitz SR, Berry WR, Gawande AA. An estimation of the global volume of surgery: a modelling strategy based on available data. *Lancet* (2008) 372:139–144.](http://paperpile.com/b/YGPhmB/wYG4j)

4. [Hall MJ, DeFrances CJ, Williams SN, Golosinskiy A, Schwartzman A. National Hospital Discharge Survey: 2007 summary. *Natl Health Stat Report* (2010)1–20, 24.](http://paperpile.com/b/YGPhmB/a74uw)

5. [Moller JT, Cluitmans P, Rasmussen LS, Houx P, Rasmussen H, Canet J, Rabbitt P, Jolles J, Larsen K, Hanning CD, et al. Long-term postoperative cognitive dysfunction in the elderly ISPOCD1 study. ISPOCD investigators. International Study of Post-Operative Cognitive Dysfunction. *Lancet* (1998) 351:857–861.](http://paperpile.com/b/YGPhmB/UrXRC)

6. [Saczynski JS, Marcantonio ER, Quach L, Fong TG, Gross A, Inouye SK, Jones RN. Cognitive Trajectories after Postoperative Delirium. *N Engl J Med* (2012) 367:30–39.](http://paperpile.com/b/YGPhmB/ipt2K)

7. [NICE. Delirium: prevention, diagnosis and management | Guidance | NICE. *NICE*](http://paperpile.com/b/YGPhmB/XLxQg) <https://www.nice.org.uk/guidance/cg103> [[Accessed October 28, 2021]](http://paperpile.com/b/YGPhmB/XLxQg)

8. [Inouye SK, Bogardus ST Jr, Charpentier PA, Leo-Summers L, Acampora D, Holford TR, Cooney LM Jr. A multicomponent intervention to prevent delirium in hospitalized older patients. *N Engl J Med* (1999) 340:669–676.](http://paperpile.com/b/YGPhmB/FZcGe)

9. [American Psychiatric Association. *Diagnostic and Statistical Manual of Mental Disorders*. American Psychiatric Association (2013). -1 p.](http://paperpile.com/b/YGPhmB/9XO7q)

10. [Mashour GA, Woodrum DT, Avidan MS. Neurological complications of surgery and anaesthesia. *Br J Anaesth* (2015) 114:194–203.](http://paperpile.com/b/YGPhmB/Bws4y)

11. [Marcantonio ER, Goldman L, Orav EJ, Cook EF, Lee TH. The association of intraoperative factors with the development of postoperative delirium. *Am J Med* (1998) 105:380–384.](http://paperpile.com/b/YGPhmB/aUNwV)

12. [Marcantonio ER, Goldman L, Mangione CM, Ludwig LE, Muraca B, Haslauer CM, Donaldson MC, Whittemore AD, Sugarbaker DJ, Poss R. A clinical prediction rule for delirium after elective noncardiac surgery. *JAMA* (1994) 271:134–139.](http://paperpile.com/b/YGPhmB/EBNPA)

13. [Gottesman RF, Grega MA, Bailey MM, Pham LD, Zeger SL, Baumgartner WA, Selnes OA, McKhann GM. Delirium after coronary artery bypass graft surgery and late mortality. *Ann Neurol* (2010) 67:338–344.](http://paperpile.com/b/YGPhmB/OsXIE)

14. [Brouquet A, Cudennec T, Benoist S, Moulias S, Beauchet A, Penna C, Teillet L, Nordlinger B. Impaired mobility, ASA status and administration of tramadol are risk factors for postoperative delirium in patients aged 75 years or more after major abdominal surgery. *Ann Surg* (2010) 251:759–765.](http://paperpile.com/b/YGPhmB/v0D4a)

15. [Patti R, Saitta M, Cusumano G, Termine G, Di Vita G. Risk factors for postoperative delirium after colorectal surgery for carcinoma. *Eur J Oncol Nurs* (2011) 15:519–523.](http://paperpile.com/b/YGPhmB/uVprT)

16. [Bruce AJ, Ritchie CW, Blizard R, Lai R, Raven P. The incidence of delirium associated with orthopedic surgery: a meta-analytic review. *Int Psychogeriatr* (2007) 19:197–214.](http://paperpile.com/b/YGPhmB/RgLNq)

17. [Tobar A E, Abedrapo M M, Godoy C J, Romero P C. Delirium postoperatorio: Una ventana hacia una mejoría de la calidad y seguridad en la atención de pacientes quirúrgicos. *Rev Chil Cir* (2012) 64:297–305.](http://paperpile.com/b/YGPhmB/IUAbX)

18. [Radtke FM, Franck M, Lendner J, Krüger S, Wernecke KD, Spies CD. Monitoring depth of anaesthesia in a randomized trial decreases the rate of postoperative delirium but not postoperative cognitive dysfunction. *Br J Anaesth* (2013) 110 Suppl 1:i98–105.](http://paperpile.com/b/YGPhmB/z1x6g)

19. [Aldecoa C, Bettelli G, Bilotta F, Sanders RD, Audisio R, Borozdina A, Cherubini A, Jones C, Kehlet H, MacLullich A, et al. European Society of Anaesthesiology evidence-based and consensus-based guideline on postoperative delirium. *Eur J Anaesthesiol* (2017) 34:192–214.](http://paperpile.com/b/YGPhmB/Ty2Dk)

20. [Chan MTV, Cheng BCP, Lee TMC, Gin T, CODA Trial Group. BIS-guided anesthesia decreases postoperative delirium and cognitive decline. *J Neurosurg Anesthesiol* (2013) 25:33–42.](http://paperpile.com/b/YGPhmB/TkOzl)

21. [Marcantonio ER, Flacker JM, Wright RJ, Resnick NM. Reducing delirium after hip fracture: a randomized trial. *J Am Geriatr Soc* (2001) 49:516–522.](http://paperpile.com/b/YGPhmB/p4TfB)

22. [Álvarez EA, Garrido MA, Tobar EA, Prieto SA, Vergara SO, Briceño CD, González FJ. Occupational therapy for delirium management in elderly patients without mechanical ventilation in an intensive care unit: A pilot randomized clinical trial. *J Crit Care* (2017) 37:85–90.](http://paperpile.com/b/YGPhmB/b8oXl)

23. [Boutron I, Altman DG, Moher D, Schulz KF, Ravaud P, CONSORT NPT Group. CONSORT Statement for Randomized Trials of Nonpharmacologic Treatments: A 2017 Update and a CONSORT Extension for Nonpharmacologic Trial Abstracts. *Ann Intern Med* (2017) 167:40–47.](http://paperpile.com/b/YGPhmB/4F2IU)

24. [Hoffmann TC, Glasziou PP, Boutron I, Milne R, Perera R, Moher D, Altman DG, Barbour V, Macdonald H, Johnston M, et al. Better reporting of interventions: template for intervention description and replication (TIDieR) checklist and guide. *BMJ* (2014) 348:g1687.](http://paperpile.com/b/YGPhmB/ZcWDA)

25. [Cruz Rivera S, Liu X, Chan A-W, Denniston AK, Calvert MJ, SPIRIT-AI and CONSORT-AI Working Group. Guidelines for clinical trial protocols for interventions involving artificial intelligence: the SPIRIT-AI extension. *Lancet Digit Health* (2020) 2:e549–e560.](http://paperpile.com/b/YGPhmB/FtlaA)

26. [MINSAL. Problema de salud AUGE N°37 Accidente Cerebrovascular Isquémico en personas de 15 años y más. MINSAL (2013).](http://paperpile.com/b/YGPhmB/mZ0v7) <https://www.minsal.cl/portal/url/item/7222754637e58646e04001011f014e64.pdf>

27. [Oh H, Seo W. Sensory stimulation programme to improve recovery in comatose patients. *J Clin Nurs* (2003) 12:394–404.](http://paperpile.com/b/YGPhmB/zBbzD)

28. [Clavet H, Hébert PC, Fergusson D, Doucette S, Trudel G. Joint contracture following prolonged stay in the intensive care unit. *CMAJ* (2008) 178:691–697.](http://paperpile.com/b/YGPhmB/cxK4s)

29. [Schweickert WD, Pohlman MC, Pohlman AS, Nigos C, Pawlik AJ, Esbrook CL, Spears L, Miller M, Franczyk M, Deprizio D, et al. Early physical and occupational therapy in mechanically ventilated, critically ill patients: a randomised controlled trial. *Lancet* (2009) 373:1874–1882.](http://paperpile.com/b/YGPhmB/lzCp2)

30. [Hashem MD, Nelliot A, Needham DM. Early Mobilization and Rehabilitation in the ICU: Moving Back to the Future. *Respir Care* (2016) 61:971–979.](http://paperpile.com/b/YGPhmB/rTuII)

31. [Taekema DG, Gussekloo J, Maier AB, Westendorp RGJ, de Craen AJM. Handgrip strength as a predictor of functional, psychological and social health. A prospective population-based study among the oldest old. *Age Ageing* (2010) 39:331–337.](http://paperpile.com/b/YGPhmB/zWD7r)

32. [Abbasi M, Mohammadi E, Sheaykh Rezayi A. Effect of a regular family visiting program as an affective, auditory, and tactile stimulation on the consciousness level of comatose patients with a head injury. *Jpn J Nurs Sci* (2009) 6:21–26.](http://paperpile.com/b/YGPhmB/BTGi6)

33. [Wei LA, Fearing MA, Sternberg EJ, Inouye SK. The Confusion Assessment Method: a systematic review of current usage. *J Am Geriatr Soc* (2008) 56:823–830.](http://paperpile.com/b/YGPhmB/6osiQ)

34. [Quiroga L P, Albala B C, Klaasen P G. Validación de un test de tamizaje para el diagnóstico de demencia asociada a edad, en Chile. *Rev méd Chile* (2004) 132:467–478.](http://paperpile.com/b/YGPhmB/qlaTr)

35. [Young Y, Fan M-Y, Hebel JR, Boult C. Concurrent validity of administering the functional independence measure (FIM) instrument by interview. *Am J Phys Med Rehabil* (2009) 88:766–770.](http://paperpile.com/b/YGPhmB/egezM)
