# Supplementary material for: Non-pharmacological prevention of postoperative delirium by occupational therapy teams: A randomized clinical trial
Source: Front Med (Lausanne). 2023 Feb 2;10:1099594. doi: 10.3389/fmed.2023.1099594 (PMC9931896; doi:10.3389/fmed.2023.1099594)
Supplement: Supplementary file 2 [file Table_2.DOCX]

**Supplemental 2. List of major surgeries.**

| MAJOR SURGERIES |
| --- |
| Hip replacement |
| Knee replacement |
| Major spine surgery |
| Esophagectomy |
| Gastrectomy |
| Pancreatectomy (total and partial) |
| Pancreaticoduodenectomy |
| Hepatectomy (lobectomy and segmentary) |
| Splenectomy |
| Colectomy |
| Hemicolectomy |
| Sigmoidectomy |
| Anterior rectal resection |
| Nephrectomy |
| Open prostatectomy surgery |
| Radical prostatectomy |
| Cystectomy |
| Mediastinal tumors |
| Lung lobectomy |
| Lung resection |
