# Supplementary material for: Non-pharmacological prevention of postoperative delirium by occupational therapy teams: A randomized clinical trial
Source: Front Med (Lausanne). 2023 Feb 2;10:1099594. doi: 10.3389/fmed.2023.1099594 (PMC9931896; doi:10.3389/fmed.2023.1099594)
Supplement: Supplementary file 3 [file Table_3.DOCX]

**Supplement 3**

**Standard Non-Pharmacological Prevention of Delirium:**

Standard non-pharmacological strategies are the first-line approach in the prevention of delirium and this type of prevention is recommended by experts in hospitalized elderly adults. Currently, studies on the subject support education and training as an important prevention strategy. The following measures were implemented for our protocol:

1. **Reorientation protocol:** performed by the nursing team, consisting of directly informing the patient at least 3 times a day of the time, date, place, and reason for hospitalization.
2. **Early mobilization:** performed by a physical therapy team twice a day. It includes a sequence of passive and active mobilization of limbs, rolling and supine activation, supine to sitting transference, sitting, sitting to standing transference, walking in place, and ambulation.
3. **Sensory deficit correction**, it encourages the use of correctors and technical aids such as glasses, hearing aids, and dentures, among others. For this, the nursing team will request the necessary implements from the family upon admission to the hospital and the patient will be reminded daily of their use.
4. **Environmental management:** installation of a clock and other orientation elements in the patient´s room to promote orientation, in addition to minimizing environmental stressors.
5. **Sleep protocol:** lowering of lights, noise, and administration of nighttime drugs.
6. **Hydration protocol:** monitoring of the patient's hydration and access to it.
7. **Reduction of medication:** mainly anticholinergics and minimization of the use of benzodiazepines.

**Table. Summary of standard delirium prevention protocol**

| **Action** | **Procedure** | **Responsible** | **Frequency** |
| --- | --- | --- | --- |
| **1. Reorientation protocol** | Directly inform the patient of the time, day, place, and reason for hospitalization. | Team of nurses | 3 times a day |
| **2. Early mobilization** | Perform motor and respiratory exercises. | Physical therapy team | 2 times a day |
| **3.Correction of sensory deficits** | Encourage the use of correctors and technical aids (glasses, hearing aids, dentures). | Team of nurses, family | 1 time per day |
| **4. Management of environment** | Install clock and other orientation elements in the patient´s room. Minimize environmental stressors. Avoid using physical restraints, replacing them with the company of family members or another member of the team. | Team of nurses, healthcare team who is treating the patient, family members | 1 time per day |
| **5. Sleep protocol** | Train the team in reducing lights, noise, and administration of drugs.  Promote night rest and daytime activation. | Team of nurses and family. | 1 time a day |
| **6. Hydration protocol** | Monitor patient hydration and check indications / availability. | Team of nurses | 2 times a day |
| **7. Decrease of medication** | Manage polypharmacy, decrease drugs with anticholinergic potential, and minimize the use of benzodiazepines. | Healthcare team who is treating the patient | 1 time a day |
