# Supplementary material for: Non-pharmacological prevention of postoperative delirium by occupational therapy teams: A randomized clinical trial
Source: Front Med (Lausanne). 2023 Feb 2;10:1099594. doi: 10.3389/fmed.2023.1099594 (PMC9931896; doi:10.3389/fmed.2023.1099594)
Supplement: Supplementary file 4 [file Table_4.DOCX]

**Supplement 4**

**Early and intensive Occupational Therapy (OT):**

Regarding OT intervention, specific training must also be carried out for the group of occupational therapists, to properly implement the OTs´ own strategies.

The OT intervention is implemented for 5 consecutive days, 2 times a day, in sessions before noon, and sessions after noon, and each session lasts 30 minutes.

The interventions to be carried out are grouped into the following 6 prevention areas:

1. **Polysensory stimulation** consists of providing the patient with intense external stimulation regulated by different sensory channels (visual, auditory, tactile, proprioceptive, and gustatory), with the objectives of increasing alertness, preventing sensory deprivation, and increasing interaction of the patient with the objects and people around him/her. Sensory stimulation protocols have proven to be effective in other pathologies, such as states of minimal consciousness and dementia. States that share some elements with delirium, therefore, can be used in patients with POD.
2. **Positioning:** consists of the early installation of orthosis and adaptations that leave areas with the highest frequency of bedsores free of pressure (sacral region, heels, among others). Proper positioning of the patient makes it possible to reduce the appearance of joint stiffness, which generates a positive impact on the patient's functionality in activities of daily living and, therefore, a reduction in the burden for health providers and their family. They also reduce the appearance of pressure ulcers, one of the conditions that causes longer hospital stays and higher costs, and finally, it allows to provide comfort to the patient during hospitalization.
3. **Cognitive stimulation**: intervention aimed at keeping mental functions active. According to the International Classification of Functionality these are: consciousness, orientation, attention, memory, calculation, praxis, and language, which are included in 6 areas of action: i) attention and wakefulness, ii) visual perception, iii) memory, iv) calculation and problem solving, v) praxis and vi) language.
4. **Basic Training in** **Activities of Daily Living (ADL)**: The intervention will focus on encouraging ADL such as of hygiene, grooming and nutrition performed independently. There are many benefits of ADL training in hospitalized elderly adults. These allow to structure their time (generating daily routines that favor the maintenance of habits), maintain their independence (functional maintenance in the daily activities of the elderly person prevents and reduces their physical, psychological, and social deterioration) and promote the feeling of usefulness. Each patient must maintain a daily routine of activities of daily living with times for cleaning, hygiene, breakfast, lunch, tea, and dinner, in a normal environment (sitting on the side of the bed or out of bed). Independent performance will be encouraged, and the occupational therapist, nurse, nursing technician or family member will only be a guide for the safe execution of the activity. Information will be provided to family members with the necessary recommendations.
5. **Motor stimulation of upper limbs:** it consists of maintaining or activating functional movements and the strength of the upper extremities, through the performance of movements towards the midline with the use of objects, bimanual coordination, ergo therapies, prehensions, writing and use of therapeutic tools. The motor stimulation of upper extremities will be used as the necessary baseline for the optimal performance of the ADL, since it has been documented that the grip force is a good predictor of independence in the ADL, cognitive status, and mortality in elderly people.
6. **Participation of family:** the incorporation of the family in health interventions, particularly with the elderly, is a feasible and effective resource, as reported in some studies. Therefore, their participation in this intervention is essential. It will begin with the identification of the family members who will collaborate with the intervention, who will be given information detailing their specific role. Likewise, they will be asked for material to work with, such as photographs or other significant elements for the elderly person.

**Table. Summary of occupational therapy protocol.**

| **Action** | **Procedure** | **Responsible** | **Frequency** |
| --- | --- | --- | --- |
| **Polysensory stimulation** | Auditory, tactile, proprioceptive, vestibular, olfactory, and visual stimulation | Occupational therapist | Patient who is on level 1, 2 or 3, according to the Riker Sedation-Agitation Scale Scale |
| **Positioning** | Evaluate according to conditions of movement and rest  Achieve a sitting position in bed, on the edge of the bed or armchair | Occupational therapist | 2 times a day |
| **Cognitive stimulation** | Encourage the use of correctors and technical aids (glasses, hearing aids, dentures). | Occupational therapist | 1 time a day |
| **Stimulation of upper limbs** | Install clock and other orientation elements in the patient´s room. Minimize environmental stressors. Avoid using physical restraints, replacing them with the company of family members or another member of the team. | Occupational therapist | 1 time a day |
| **Training in Activities of Daily Living (ADL)** | Training in ADL, sitting on the edge of bed or in an armchair, feeding, modified bathing, transfers or changing room can be performed | Occupational therapist | 1 time a day |
| **Family** | Delirium education and family stimulation | Occupational therapist | 2 times a week |
